# AI-Enhanced FT-IR Spectroscopy: Evaluation of a Novel Tool for High-Throughput Serovar Typing of *Salmonella enterica* subsp. *enterica* in Croatia

**DOI:** 10.3390/pathogens14090856

**Published:** 2025-08-28

**Authors:** Maja Dopuđ, Sandra Šuto, Dora Tomašković, Lucija Hlebić, Lovran Peinović, Silvio Špičić, Maja Zdelar Tuk, Irena Reil, Andrea Humski, Gordan Kompes, Silvija Šoprek Strugar, Bojan Papić, Jana Avberšek, Andrzej Mikolajczak, Sanja Duvnjak

**Affiliations:** 1 Laboratory for Bacterial Zoonoses and Molecular Diagnostics of Bacterial Diseases, Department for Bacteriology and Parasitology, Croatian Veterinary Institute, 10000 Zagreb, Croatia; dopud@veinst.hr (M.D.); spicic@veinst.hr (S.Š.); zdelar-tuk@veinst.hr (M.Z.T.); marjanovic@veinst.hr (S.D.); 2 Department for Respiratory System Infections, and Skin and Soft Tissue Infections, Clinical Microbiology Service, Andrija Štampar Teaching Institute of Public Health, Clinical Microbiology Service, 10000 Zagreb, Croatia; sandra.suto@stampar.hr; 3 Laboratory for Food Microbiology, Department for Veterinary Public Health, Croatian Veterinary Institute, 10000 Zagreb, Croatia; stojevic@veinst.hr (D.T.); hlebic@veinst.hr (L.H.); peinovic@veinst.hr (L.P.); humski@veinst.hr (A.H.); 4 Laboratory for General Bacteriology and Mycology, Department for Bacteriology and Parasitology, Croatian Veterinary Institute, 10000 Zagreb, Croatia; kompes@veinst.hr; 5 University Hospital for Infectious Diseases Dr. Fran Mihaljević, 10000 Zagreb, Croatia; ssoprek@bfm.hr; 6 Institute for Microbiology and Parasitology, Veterinary Faculty, University of Ljubljana, 1000 Ljubljana, Slovenia; bojan.papic@vf.uni-lj.si (B.P.); jana.avbersek@vf.uni-lj.si (J.A.); 7 Microbiology & Infection Diagnostics Division, Eastern Europe, Bruker Daltonics GmbH & Co. KG, 28359 Bremen, Germany; andrzej.mikolajczak@bruker.com

**Keywords:** AI, FT-IR spectroscopy, IR Biotyper, *Salmonella enterica*, one health

## Abstract

Rapid and accurate serotyping of *Salmonella* (*S.*) *enterica* subsp. *enterica* serovars is essential for effective public health surveillance, outbreak control, and food safety management. Traditional serotyping, although considered the gold standard, is time-consuming, technically demanding, and costly. This study aimed to evaluate the applicability of artificial intelligence (AI)-enhanced Fourier-transform infrared (FT-IR) spectroscopy using an IR Biotyper (Bruker Daltonics, Bremen, Germany) for the rapid and accurate serotyping of *Salmonella enterica* subsp. *enterica* isolates in Croatia. Materials and Methods: A total of 143 isolates representing different *S. enterica* serovars of human and food origin were analysed using the IR Biotyper. Each strain was tested in three biological and at least three technical replicates. The obtained results were compared with traditional serotyping according to the Kauffmann–White–Le Minor scheme. Isolate identification at the genus level was further confirmed by MALDI-TOF mass spectrometry. Results: The IR Biotyper demonstrated high reproducibility and complete concordance with standard serotyping methods, enabling accurate differentiation of the most prevalent *S. enterica* serovars in Croatia. Conclusions: Our findings demonstrate the applicability of FT-IR in routine laboratory work, with the potential to reduce typing time, reduce the number of strains, and lower overall costs required for epidemiological surveillance within the One Health approach.

## 1. Introduction

Infections with *Salmonella* (*S.*) *enterica* subsp. *enterica* serotypes are still considered Europe’s second-most prevalent foodborne gastrointestinal zoonosis. Despite the integration of farm-to-fork control programmes, it continues to pose a significant public health burden in both resource-limited and developed countries across Europe [1,2,3].

Based on the polysaccharide portion of the lipopolysaccharide layer (O-antigen) and/or the filamentous portion of the flagella (H-antigen) presented on the surface of bacteria, there are more than 2600 serovars and around 50 serogroups that *S. enterica* encompasses [4,5,6]. Various *S. enterica* serovars exhibit differences in their pathogenicity, virulence, and susceptibility to antimicrobial treatment. Therefore, a wide variety of *S. enterica* serovars have the potential to cause infections in both humans and animals. However, only a limited number of these serovars are responsible for most human diseases, with many serotypes being host-specific or unable to cause infection in humans [5,7,8]. The severity of the infection depends on the specific combination of the infectious dose of the involved *Salmonella* serotype and the host’s immune status, with the infection classified into two types: typhoidal and non-typhoidal [2,9,10].

As a zoonotic disease, *Salmonella* outbreaks are often linked to the consumption of contaminated food products, including poultry, poultry products, cattle, and dairy products. However, more recently, an increasing number of outbreaks have been associated with contaminated fruits and fresh vegetables. By this means, it is connected with both endemic and epidemic scenarios [2,11].

According to the European Food Safety Authority (EFSA), approximately 91,000 human salmonellosis cases are reported annually in the European Union (EU) [6,12]. In Croatia alone, the number of confirmed cases in 2022 was 1047, resulting in a rate of 27.1 cases per 100,000 population. That makes Croatia one of the top five countries with the highest notification rates of human salmonellosis in the EU. While common *Salmonella* serovars remain a persistent public health issue, Croatia has also reported cases of rare *Salmonella* strains, such as *S.* Mikawasima. The Mikawasima serovar was involved in a significant outbreak in neonatal and maternal wards at the University Hospital of Split, becoming endemic until the end of 2024. The outbreak was characterised by the presence of extended-spectrum beta-lactamase (ESBL)-producing *S*. Mikawasima in stool. *S*. Mikawasima is a common cause of hospital-acquired infections and is usually present in animals and contaminated environmental and food samples. These occurrences highlight the importance of continuous surveillance and rapid response strategies to prevent the spread of both common and rare *Salmonella* serovars [13].

Also in 2022, the most frequently isolated serovars in the EU were *S*. Enteritidis (54.6%), *S*. Typhimurium (11.4%), and a monophasic variant of *S*. Typhimurium (8.8%), whereas *S*. Enteritidis is commonly associated with poultry and products, while *S*. Typhimurium has a wider species range, including pigs and cattle as well as poultry [12,14]. Other notable serovars included *S*. Infantis (2.0%) and *S*. Derby (0.93%), with *S*. Derby being closely followed by *S*. Coeln (0.91%) [14].

Differentiation of *S. enterica* subsp. *enterica* at the subspecies/serovar level is crucial for epidemiological studies, managing foodborne outbreaks, vaccine development, disease detection, and treatment modality decisions [15]. For that reason, a wide variety of typing methods have been used for the identification and differentiation. Among them, serotyping based on the agglutination reaction with specific antisera targeting the somatic O-antigen and flagellar H-antigens is the most widely used method for classifying *S. enterica* serovars (Kauffmann–White–Le Minor scheme). Nevertheless, the method is time-consuming, labour-intensive, requires experience and specialised equipment, and is highly expensive [2,16]. On the other hand, whole-genome sequencing (WGS) has become the gold-standard method for bacterial typing due to its higher discriminatory power and increased accessibility. It is used for outbreak investigations and targeting control measures, and allows the detection of virulence factors and antimicrobial resistance genes. Hence, it is expensive, time-consuming, and technically demanding [17,18,19]. Additionally, MALDI-TOF MS (matrix-assisted laser desorption/ionisation time-of-flight mass spectrometry) has been employed to identify *Salmonella*. However, it is unsuitable for identifying *Salmonella* on the serovar level [20]. Therefore, there is always a need to develop accurate and rapid strain typing methodologies, which will be indispensable for the effective management and control of infections and other related challenges in routine work.

Fourier-transform infrared spectroscopy (FT-IR) has been used in analytical chemistry for decades to determine the chemical composition of a wide range of samples. FT-IR has been successfully used for the discrimination and classification of bacteria at different taxonomic levels, from genera down to strain level, based on the differences in the infrared absorption patterns of microbial cells and/or outer membrane cell components (carbohydrates, lipids, nucleic acids, protein and lipopolysaccharides) [2,3,21,22,23,24]. Due to variations in the cell’s composition and structure, absorption will differ. Therefore, FT-IR will produce unique fingerprint-like signatures for each microbial cell, which can be compared to other spectra using hierarchical or non-hierarchical clustering to investigate their similarity [24,25,26]. For this purpose, machine learning algorithms have been employed, including artificial neural networks (ANNs) and support vector machines (SVMs), as well as an outlier detector [26,27].

Due to its low analytical costs, short per-sample hands-on time, high throughput, rapidity, and cost-effectiveness, FT-IR is rapidly expanding in research areas, especially in the investigation of *Salmonella* microorganisms [2,25,26,28]. Since *S. enterica* exhibits high antigenic diversity and varying clinical relevance, it has been an ideal candidate for studying with Fourier-transform infrared (FT-IR) spectroscopy. In addition to the different lengths of somatic antigens and the high diversity of carbohydrate composition in O-units, which contribute to the surface cell structure, this enables greater differentiation based on FT-IR methodology [20]. Several studies have been conducted and shown that FT-IR has high discriminatory power, enabling differentiation at the serogroup/serovar level [1,2,3,16,20,21,29].

In this study, our main objective was to analyse a collection of the most frequent and clinically relevant *S. enterica* subsp. *enterica* isolates. We aimed to determine the discriminatory power of FT-IR using IR Biotyper (Bruker Daltonics, Bremen, Germany) in combination with multivariate data analysis and MALDI-TOF MS to identify and discriminate different *S. enterica* subsp. *enterica* serovars accurately. The ultimate goal was to provide evidence for the potential application of FT-IR diagnostics on clinical pathogenic samples.

## 2. Materials and Methods

### 2.1. Bacterial Collection

The isolates were obtained from the microbial culture collection of the Laboratory of Food Microbiology, established at the Croatian Veterinary Institute in Zagreb, Croatia, and originated from human and food samples.

A total of 143 isolates of *Salmonella enterica* subsp. *enterica* of human and food-related origins were analysed by FT-IR. The collection of investigated isolates included the most common *Salmonella* O-serogroups, such as O:4, O:7, O:8, and O:9. These serogroups comprise nine clinically and epidemiologically relevant serovars: *S*. Enteritidis, *S*. Typhimurium, and monophasic *S*. Typhimurium, which are among the most frequently reported in Croatia and worldwide. Additionally, others considered in the dataset were *S*. Derby, *S*. Infantis, *S*. Mbandaka, *S*. Hadar, and *S*. Coeln.

All isolates were previously confirmed at the serovar level by traditional serotyping (HRN EN ISO-6579-1:2017/A1:2020) at the Laboratory of Food Microbiology, Croatian Veterinary Institute (Kauffmann–White–Le Minor scheme). The laboratory serves as the national reference laboratory for salmonellosis and holds accreditation for the applied method.

Additionally, all isolates were confirmed at the genus level by MALDI-TOF MS as *Salmonella* spp. The MALDI-TOF MS analysis was performed on the MALDI-Biotyper system, which consists of an LT microflex mass spectrometer (Bruker Daltonics, Bremen, Germany).

To evaluate the specificity and reliability of the classifier, a set of 30 non-*Salmonella* isolates, such as *Escherichia coli*, *Corynebacterium pseudotuberculosis*, and *Staphylococcus aureus*, was included in the analysis. These isolates served as negative controls, allowing assessment of the model’s ability to differentiate non-*Salmonella* species from *S. enterica* subsp. *enterica* strains. This step was essential to ensure that the classifier did not misclassify unrelated bacterial species as *Salmonella*, thereby validating its reliability and specificity. The isolates were prepared the same way as *S. enterica* subsp. *enterica* strains, following the manufacturer’s instructions [30].

### 2.2. Strain Preparation

Pure and selected bacterial strains were cultured on blood agar medium (Blood Agar Base No. 2 (Oxoid, Hampshire, UK) with 5% defibrinated blood and 1% esculin, adjusted to pH 7.4 ± 0.2 at 25 °C). All isolates were cultured in three independent biological replicates to obtain more comprehensive spectra for each serovar. The biological replicates of each strain were three subcultures from the original “mother” plate cultured on the blood agar medium for 22 ± 2 h at 37 °C. When subculturing, no specific colony selection criteria were applied beyond ensuring that the selected colonies were single and pure.

IRBT sample preparation was carried out in accordance with the manufacturer’s instructions using the IR Biotyper kit (Bruker Daltonics, Bremen, Germany) [30]. Three loops of bacterial cells were resuspended in 50 μL of freshly prepared 70% ethanol (EMSURE Ethanol, absolute (Merck, Rathway, NJ, USA)) solution in 1.5 mL tubes filled with beads supplied in the manufacturer’s kit (IR Biotyper kit, Bruker Daltonics, Bremen, Germany). After vortexing, 50 μL of sterile Milli-Q water (in-house) was added, and the solution was vortexed. The tubes were briefly spun down in a centrifuge to avoid cross-contamination. Before placing 15 μL of each bacterial suspension on one spot of a silicon IR Biotyper plate, each sample was mixed by pipetting to ensure that a homogenous solution was being analysed. The first three rounds of 30 samples were tested in three technical replicates. In the last two rounds of samples, each sample was tested in five technical replicates. The silicon plate with suspensions was dried for 30 min at room temperature before being placed in the IR Biotyper for the reading.

The quality control InfraRed Test Standards (IRTS 1 and IRTS 2) of the IR Biotyper kit (Bruker Daltonics, Bremen, Germany) were used as quality controls prior to sample spectra acquisition in each run. IRTS1 and IRTS2 were prepared according to the manufacturer’s instructions. First, the standards were resuspended in 100 μL of deionised water. After extensive vortexing, the suspension was incubated for 30 min in a thermomixer (Eppendorf, Hamburg, Germany) at approximately 1,500 rpm at room temperature. Afterwards, 60 μL of absolute ethanol was added and gently mixed by pipetting, followed by brief vortexing. Ten μL of each IRTS was spotted in duplicate onto two spots of the IR Biotyper silicon plate and left to dry as described for the samples. Additionally, one blank spot was left on the IR Biotyper silicon plate as a background control.

### 2.3. Spectra Acquisition and Analysis

The IR Biotyper spectrometer measures absorption spectra in transmission mode in the spectral range of 4000–500 cm^−1^ (mid-IR). The silicon plate with dried suspensions of samples and controls was inserted into the instrument. The instrument operates under ambient environmental conditions without specific purge requirements (e.g., no controlled humidity or gas purge system is employed).

After measurement, the resulting spectra are subjected to a Quality Test (QT) by the instrument’s software (IR Biotyper^®^ Client software V3.0, Bruker Daltonics, Bremen, Germany). Each spectrum, including both background and sample measurements, was acquired with 32 scans. Spectra of poor quality (inadequate minimum and maximum absorbance values and signal-to-noise ratio) were excluded. In contrast, spectra with acceptable quality were used for further analyses. After smoothing the spectra with the Savitzky–Golay algorithm, the second derivative was calculated over 9 datapoints. The purpose of the Savitzky–Golay algorithm is to smooth and denoise the spectrum in FT-IT for the multicomponent hydrocarbon spectrum reconstruction in the mixed gas spectra analysis. The main performance index of the Savitzky–Golay smoothing filter was determined by the polynomial order and frame size when the Savitzky–Golay filter was used for smoothing. The spectra were then cut to the relevant spectral window of 1300–800 cm^−1^ (the oligo- and polysaccharide region). Finally, all spectra were vector-normalised to account for preparation-related variance in biomass and hence absorption [3,31]. All qualitatively acceptable spectra were classified using the *Salmonella* O-groups v3 classifier integrated into the IR Biotyper Client software version 3.0. The serogroup classification of each spectrum was compared to its original NRL-*Salmonella* serovar allocation, and no discrepancies were recorded. An exploratory data analysis was conducted on the entire dataset using Principal Component Analysis (PCA) and Linear Discriminant Analysis (LDA) to generate 2D and 3D scatter plots, visualising the distribution of *Salmonella* serogroups and serovars. These algorithms are implemented in the IR Biotyper software. PCA provides a rapid overview and is calculated over the 501 datapoints which form the spectral region used for the analysis (1300–800 cm^−1^). On the other hand, LDA enables deeper resolution, particularly within complex serogroups, and is calculated using the first principal components that together comprise 95% of the variance. No statistical analysis was performed to assess PCA/LDA results.

## 3. Results

Accurate identification and differentiation of *Salmonella enterica* subsp. *enterica* serogroups and serovars are critical for epidemiological surveillance, food safety, and clinical diagnostics. This study assessed the performance of the IR Biotyper system in distinguishing between O-serogroups and their constituent serovars. Therefore, a single-laboratory validation study was performed on a commercially available classifier with IR Biotyper.

Through this study, a consistent spectral splicing methodology, combined with multivariate analysis (PCA and LDA), was applied, with additional support from Bruker’s integrated classifier.

### 3.1. Discrimination Between Serogroups of S. enterica *subsp*. enterica

All 1985 absorption spectra from 143 *S. enterica* subsp. *enterica* isolates were recorded and submitted for classification using the *Salmonella* O-group v3 classifier provided by Bruker Daltonics (Bremen, Germany).

For each isolate, one independent biological replicate was analysed. Each biological replicate consisted of three technical replicates, which were independently conducted and measured over three days. The region of the IR spectrum that proved to be the best for discriminating S. enterica isolates was the region from 1300 to 700 cm^−1^.

Only spectra of acceptable quality [0.4 < absorption < 2, signal/noise >40, signal/water >20, fringes (×10^−6^) < 100] were used for further analyses, while spectra of poor quality were excluded. The resulting spectra were subjected to a serogroup allocation by the classifier. The FT-IR classifier v3.0 analysed each spectrum obtained from the *Salmonella* isolate, allowing for a comparison of the resulting serogroup allocation with the known serotype to assess concordance.

**Figure 1:**
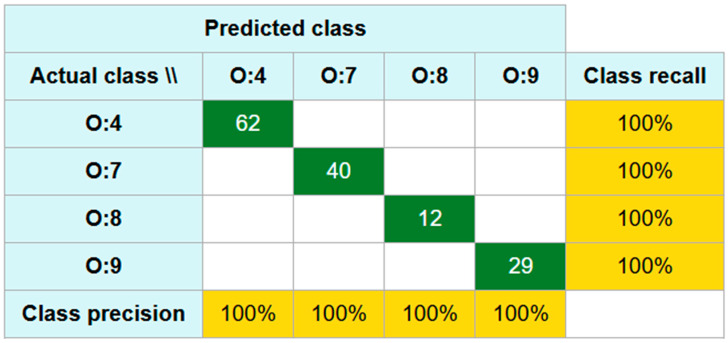
A confusion matrix illustrating the overall agreement of the classifier derived from the spectra of the tested *Salmonella enterica* subsp. *enterica* isolates. All spectra were correctly classified. The class recall (which corresponds to sensitivity) and class precision (which corresponds to specificity) are both 100% for all *Salmonella enterica* subsp. *enterica* isolates.

Out of a total n = 1985 spectra derived from n = 143 isolates, all spectra passed the quality control criteria and none were discarded. Furthermore, all spectra were correctly classified by the model, yielding a positive agreement of 100%. Similarly, the negative agreement of the negative control was 100%, indicating that all spectra originating from different sets of non-*Salmonella* isolates were correctly excluded (i.e., not misclassified as target). These results demonstrate the high discriminatory performance of the model, with both positive and negative agreement of 100% (Figure 1).

**Figure 2:**
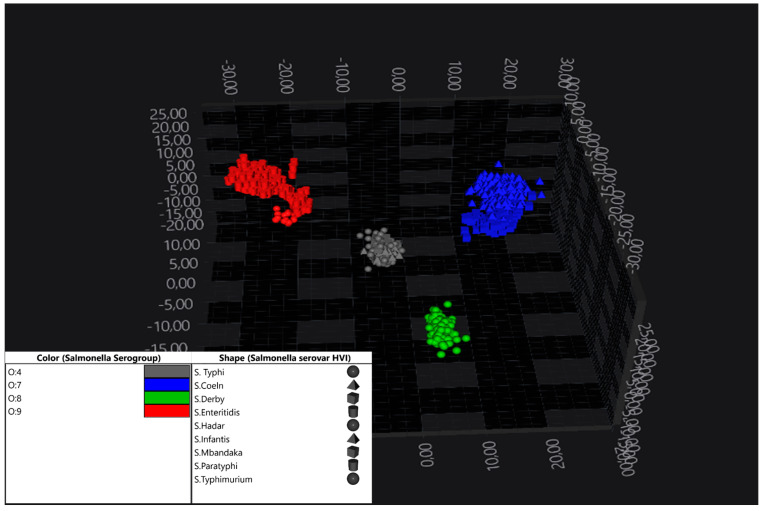
LDA 3D scatter plot showing the distribution of four O-serogroups of *S. enterica* subsp. *enterica* isolates in IR spectral space. Thirty principal components were used to create an LDA by O-groups, which improved separation. O-serogroups colour the spectra, and the shapes correspond to individual isolates. The LDA model proved to be very robust in separating the serogroups.

Using Principal Component Analysis (PCA) alone (0.95/20), a clear separation of the four O-serogroups included in the dataset was observed (Figure 2). The application of Linear Discriminant Analysis (LDA) (1.00/30) significantly improved this separation. However, while inter-group discrimination improved, the ability to resolve serovars within each group decreased when all groups were analysed simultaneously. This necessitated a targeted, group-specific (“zoom-in”) analysis for intra-group differentiation. Nevertheless, the exploratory analysis with PCA and LDA enabled clear differentiation of serogroups.

Additionally, the Bruker classifier also demonstrated effective real-time identification of serogroups during the analysis run, highlighting its potential for automated classification in routine workflows.

### 3.2. Discrimination Within S. enterica *subsp*. enterica Serogroups (Serovar Identification)

**Figure 3:**
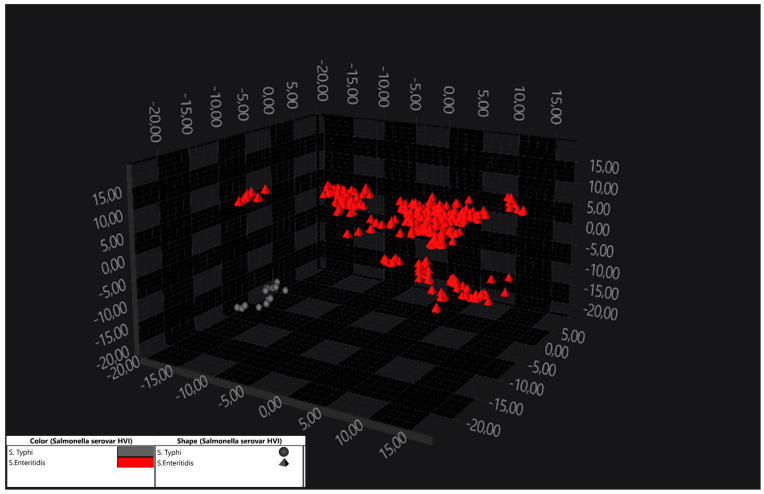
LDA 3D scatter plot showing the separation of the *S*. Enteritidis isolates (in red) from the *S*. Typhi isolates (in grey). LDA was performed based on variance and by assessing the isolates as a group identifier. Each point represents the spectrum of one strain.

The O:9 serogroup included one *S*. Typhi isolate and multiple *S*. Enteritidis strains. With LDA (1.00/30), a clear separation of these two serovars was enabled. In the O:9 serogroup, only one *S*. Typhi isolate was available, yet it was distinctly separated from *S*. Enteritidis strains, as seen in Figure 3.

**Figure 4:**
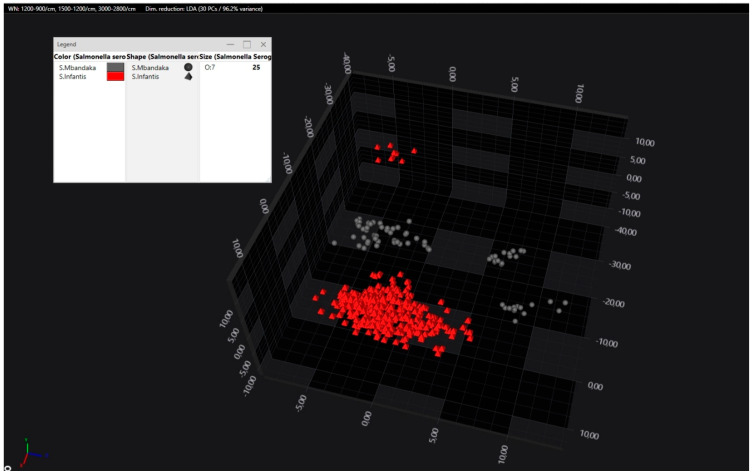
LDA of the O:7 serogroup. *S*. Infantis (red colour) isolates are separated from *S*. Mbandaka (grey colour). Each point represents the spectrum of one strain.

Most isolates within the O:7 group were separated reliably. While some overlaps were observed in scatter plots, deviation plots using multiple LD planes (e.g., LD1 vs. LD2 vs. LD3 or LD5) revealed clear separation (Figure 4).

The O:8 serogroup comprised only the *S*. Hadar serovar and was resolved. This finding demonstrates a good baseline for group homogeneity. A larger number of isolates within this group should be considered for future investigation to confirm these observations.

**Figure 5:**
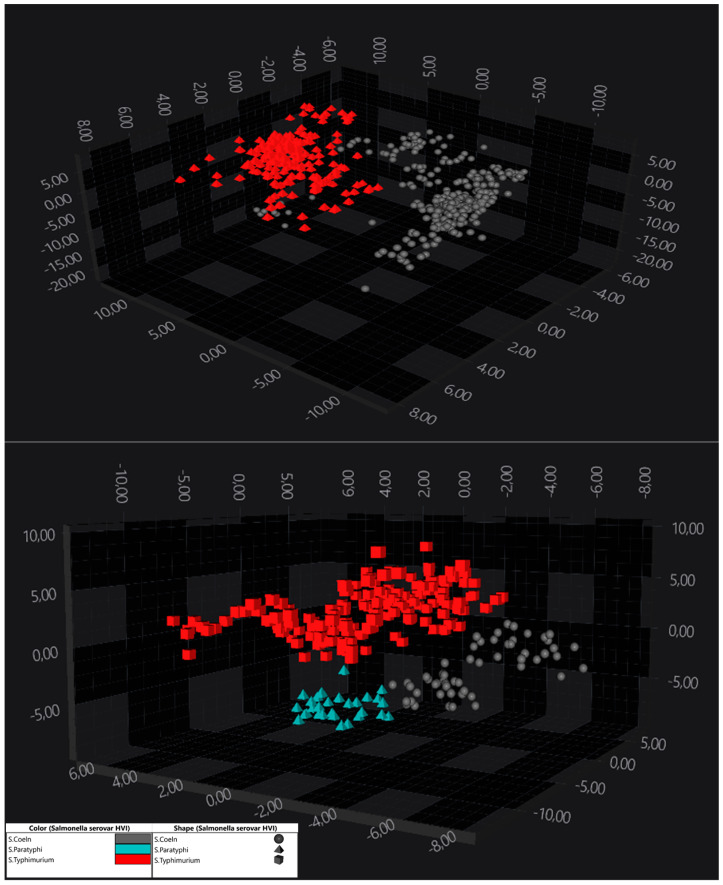
Three-dimensional scatter, showing a clear separation of serovars of *Salmonella* serogroup O:9 species in IR spectral space. The spectra are coloured according to the serovars. In the picture above, red triangles represent *S*. Typhimurium, while grey circles represent *S*. Derby. In the picture below, there is a clear separation of three serovars: *S*. Paratyphi (cyan triangles), *S*. Coeln (grey circles), and *S*. Typhimurium (red squares). Every symbol represents a different isolate.

The serogroup that posed the most significant challenge for serovar separation was O:4. Nevertheless, deviation plots highlighted distinct separation between serovars such as *S*. Paratyphi and *S*. Coeln from the rest in the LD2, LD3, and LD4 axes. Visual clarity was limited in 2D scatter plots, emphasising the utility of higher-dimensional projections. A refined analysis with LDA revealed two distinct groups within the O:4 group, provisionally labelled as O:4-I and O:4-II. O:4-I showed good separation between *S*. Derby and *S*. Typhimurium (Figure 5, top image), while O4-II showed perfectly separated *S*. Coeln, *S*. Paratyphi, and *S*. Typhimurium (Figure 5, bottom image).

## 4. Discussion

Accurate identification and differentiation of *Salmonella enterica* isolates is essential in clinical microbiology, veterinary medicine, and food safety. Depending on the application, *Salmonella* typing may require different levels of resolution—from serogroup or serovar determination to precise strain characterisation in outbreak settings. Therefore, there is a constant need for enhancing identification and detection methods capable of identifying and typing this pathogen and preventing outbreaks [3,25]. Conventional serotyping, although widely used, has several drawbacks. It is labour-intensive, typically requires at least two days to complete, and remains partly subjective since results depend on the skill and experience of the staff performing the assays. Moreover, the use of expensive antisera and the need to work with live *Salmonella* cultures also increase both the cost and the biosafety risks of the procedure. While high-resolution methods such as whole-genome sequencing (WGS) offer powerful discriminatory capacity, they are likewise resource-intensive and laborious. In this context, Fourier-transform infrared (FT-IR) spectroscopy is gaining attention as a promising alternative, offering speed, cost-effectiveness, and high throughput for sub-serogroup typing [3,25,26].

Therefore, this study evaluated whether AI-enhanced FT-IR spectroscopy using the IR Biotyper is suitable for rapid, accurate, and user-friendly identification and differentiation of *S. enterica* subsp. *enterica* serovars. The strain collection included the most common serovars in Croatia—*S*. Typhimurium (and its monophasic variant), *S*. Enteritidis, *S*. Typhi, *S*. Paratyphi, *S*. Infantis, *S*. Mbandaka, *S*. Coeln, *S*. Derby, and *S*. Hadar. The IR Biotyper achieved 100% overall agreement in differentiating *Salmonella* O-serogroups and serovars, with no misclassifications. These findings are consistent with previous studies [3,20,21,32,33,34,35,36] and confirm that, when combined with high-dimensional data analysis, the IR Biotyper can effectively distinguish *Salmonella* at both the serogroup and serovar levels.

Nonetheless, the study has limitations. This study focused on the most commonly isolated serovars in Croatia, many of which are also among the most prevalent in the EU. Also, the number of isolates within some serogroups was limited. For example, in the O:8 serogroup, only the *S*. Hadar serovar was available, and in the O:9 group, although there was clear separation between *S*. Typhi and *S*. Enteritidis, only a single *S*. Typhi isolate was analysed. Therefore, future work should include larger numbers of isolates overall, representatives from every serogroup, and a more diverse panel of serovars, including rare and emerging ones.

Previous reports [18,22,26,37,38,39] have shown that FT-IR can be used for real-time surveillance: for example, a 2024 study demonstrated that FT-IR correctly determined the clonal relatedness of *E. coli* isolates during an ongoing outbreak, in agreement with WGS results [40]. Another study showed that the IR Biotyper accurately clustered *Klebsiella pneumoniae* strains, producing typing results almost entirely concordant with PFGE and WGS [41]. Such evidence supports the potential of the IR Biotyper as a novel real-time typing tool for various microorganisms, including the detection of clonal spread in healthcare settings. While such evidence underscores the IR Biotyper’s potential for real-time surveillance, our study did not assess its biotyping capacities directly against WGS—the current gold standard for high-resolution typing. This comparison will be an important focus of future work.

It is also worth mentioning several factors that can affect the reproducibility in FT-IR-based typing. FT-IR spectral profiles can be affected by variations in growth medium composition, incubation time and temperature, colony purity, and sample handling steps such as ethanol concentration, bead-beating consistency, and spotting volume. These factors may introduce variability in the spectra, underscoring the importance of standardised sample preparation, data acquisition, and analysis protocols to ensure the reliability and reproducibility [27]. While strict protocols were followed in the present study to minimise these variables, broader inter-laboratory evaluations are needed to assess the robustness of the IR Biotyper system under different operating conditions. However, a very small number of laboratories have access to the IR Biotyper device, and such evaluations are being planned for future dates.

Although standardisation and broader validation are still needed, the present findings highlight important practical implications. In clinical microbiology, the IR Biotyper can reduce turnaround time for serovar identification, which would help laboratories deliver faster diagnoses and initiate outbreak control measures more quickly. In veterinary settings, applying this approach to routine monitoring of livestock and poultry could aid in detecting and controlling *Salmonella* spread before it reaches the food chain. For food safety, its capacity to analyse many isolates at low cost makes it a valuable option for surveillance laboratories and for tracing sources during suspected outbreaks. By linking applications across human, animal, and food health sectors, AI-enhanced FT-IR spectroscopy aligns with the principles of the One Health framework.

## 5. Conclusion

This study demonstrates that AI-enhanced FT-IR spectroscopy using the IR Biotyper (Bruker Daltonics, Germany) is a reliable and highly discriminatory method for serotyping *Salmonella enterica* subsp. *enterica*. It achieved complete agreement with conventional serotyping and MALDI-TOF MS for the most prevalent serovars in Croatia. While these results are promising, they were obtained under single-laboratory conditions with a limited number of serovars, so broader validation, including rare isolates and inter-laboratory comparisons, is required.

Given its speed, low cost, and high throughput, the method has the potential value for clinical, veterinary, and food safety applications within a One Health framework. Future work should compare FT-IR against whole-genome sequencing and evaluate its performance during real outbreak scenarios.

## Data Availability

No data created in this manuscript.
